# On the choice of linkage statistics

**DOI:** 10.1186/1753-6561-1-s1-s102

**Published:** 2007-12-18

**Authors:** Patricia Margaritte-Jeannin, Marie-Claude Babron, Françoise Clerget-Darpoux

**Affiliations:** 1 INSERM U535, F-94817 Villejuif, France; 2 Univ Paris-Sud, IFR69, UMR535, F-94817 Villejuif, France

## Abstract

Three LOD score statistics are often used for genome-wide linkage analysis: the maximum LOD score, the LOD score statistic proposed by Kong and Cox, both based on the allele-sharing between affected sib pairs, and the maximization of the LOD score function of Morton on two genetic models and an heterogeneity parameter.

Using only identity-by-descent sharing between affected sibs as linkage information, we studied the behavior of these three statistics under the null hypothesis in the rheumatoid arthritis simulated data (Genetic Analysis Workshop 15 Problem 3 – simulating model known). Distributions under the null hypothesis show that identical values of the statistics correspond to very different genome-wide *p*-values: comparison and interpretation of several linkage statistics cannot be done on the observed value. The Kong and Cox LOD score statistic had slightly better power to detect the HLA region involved in rheumatoid arthritis compared to the other methods. In a second step, we show that performing the analysis under a greater number of genetic models in the hope of better scanning the space of models, does not increase the power of detection.

## Background

Genome-wide linkage studies are often performed on affected sib pairs to detect disease susceptibility genes in multifactorial diseases. Many statistics have been proposed to achieve such a goal.

Two methods, the LOD score statistic proposed by Kong and Cox (KC-LOD) [1], which is an extension of the nonparametric linkage statistic [2], and the maximum LOD score (MLS) [3], are based on the allele sharing between affected sibs and do not require the specification of a model at the disease locus which, in the case of a multifactorial disease, is unknown.

An alternative strategy, proposed by Greenberg et al. [4], maximizes the LOD score function of Morton [5] on two genetic models at the disease locus and on an additional heterogeneity parameter. We will call this statistic HLOD-S1. With the idea of better scanning the space of models to improve power, many authors (e.g., [6-9]) considered a wider set of genetic models, without consensus on which and how many models should be employed. Here, we will focus on the maximum statistic obtained over four different genetic models, which we will call HLOD-S2.

These statistics are all LOD scores, i.e., the decimal logarithm of the ratio of two likelihoods (linkage versus no linkage). They are computed at each marker of a given chromosome, taking into account the multipoint information provided by the entire set of markers. The maximum value observed for each chromosome is then retained to perform the linkage test. However, since these statistics differ on the parameters on which the maximization is achieved, they are likely to have different statistical properties.

In this work, we study the behavior of these statistics under the null hypothesis, and then evaluate their performance for detecting the HLA risk factor in the rheumatoid arthritis (RA) simulated data (Genetic Analysis Workshop 15 Problem 3). The simulating model was known prior to the analysis.

## Methods

### Material

The segregation of 730 microsatellite markers, spaced on 22 chromosomes with an average inter-marker distance of about 5 cM, was simulated on 100 replicates of 1500 families with at least two affected sibs.

Preliminary linkage analyses showed that it was not possible to make any power comparison with such sample sizes: all linkage statistics were highly significant for detecting the role of HLA, while their power was very low (less than 5%) for the other loci. Therefore, we decided to focus on the detection of the susceptibility factor in the HLA region and to split each replicate into smaller family samples in order to have a lower, but not too low, power of detection. A sample size of 60 seemed appropriate. Each replicate was split into 25 sub-samples. The study was thus performed on 2500 replicates of 60 families each. Parental status was considered unknown in all replicates, so that linkage information consists of the identity-by-descent (IBD) sharing between affected sibs.

### Linkage statistics

The data were analyzed by four LOD score statistics, MLS, KC-LOD, HLOD-S1, and HLOD-S2.

The MLS [3] maximizes the likelihood of the IBD sharing vector, within the possible triangle constraints [10]. Under the null hypothesis, the expected IBD vector is [0.25; 0.50; 0.25]. Calculations were performed with the Mapmaker/Sibs software [11].

The KC-LOD proposed by Kong and Cox [1] is maximized on a single parameter, δ, that represents the degree of allele sharing among affected individuals. Under the null hypothesis, δ is equal to 0, and the higher δ, the higher the allele sharing. KC-LOD analysis was carried out under the "score pairs" option and the exponential model proposed by Kong and Cox with Allegro v1.2 [12].

HLOD-S1 was calculated as initially proposed by Greenberg et al. [4] under a dominant and recessive model, each with a disease allele frequency of 0.01, a penetrance of 0.50, and no phenocopies. The LOD score function was maximized over these two models and the heterogeneity parameter, α, represented the proportion of families linked to the disease locus. In HLOD-S2, two additional models were considered, with a disease allele frequency of 0.2. The LOD score function was then maximized over these four genetic models and the parameter α. All HLOD calculations were done with the Allegro v1.2 software [12].

We first studied the distribution of these four statistics under the assumption of no linkage by analyzing the 16 chromosomes that did not harbor a susceptibility gene. The maximum value on each chromosome was recorded for each statistic, leading to 40,000 values (2500 replicates × 16 chromosomes). This provides the distribution of the maximum of each statistics for an average chromosome. Thus, for a full genome scan, one may apply a Bonferroni correction for 22 chromosomes. This procedure can be used either to determine the threshold for a genome-wide type I error of 5% (nominal *p *= 0.002 per chromosome) or to determine the genome-wide *p*-value corresponding to a given value of the statistics.

The power for detecting linkage was calculated as the number of times a given statistic exceeded the threshold corresponding to a genome-wide type I error of 5%. Two loci in the HLA region were known to be involved in the manifestation of the simulated disease. We considered the HLA region to be detected if there was evidence for linkage in the 20-cM interval around the HLA-DR locus, i.e., in the interval [STRP6_10-STRP6_13].

## Results and discussion

### Distribution of the statistics under the null hypothesis

**Figure 1:**
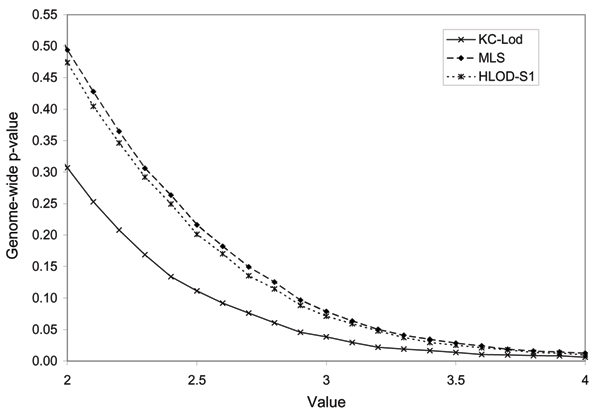
False-positive rate as a function of the observed values of three linkage statistics under no linkage.

Figure 1 shows the false-positive rates corresponding to the observed values of the KC-LOD, MLS, and HLOD-S1 under the null hypothesis. For clarity, the graph is limited to values between two and four.

Although the three linkage statistics are maximum LOD scores, their distributions are different. This is due to the different underlying parameterization. Note, however, that MLS and HLOD-S1 have very similar distributions. The HLOD-S2 statistic, performed under four different genetic models, has a distribution similar to that of MLS and HLOD-S1 (data not shown).

Identical values of observed KC-LOD and MLS (or HLOD-S1) give rise to very different genome-wide *p*-values. For example, when a value of two is observed, the false-positive rate is 31% for KC-LOD, while a value of two attains a false-positive rate of almost 50% for the MLS and HLOD-S1 (49.4 and 48.4%, respectively). This shows that the comparison and interpretation of several linkage statistics cannot be done on the observed value.

The thresholds corresponding to a genome-wide type I error of 5% are 2.89, 3.23, and 3.19 for the KC-LOD, MLS, and HLOD-S1, respectively.

### Power for detecting linkage in the HLA region

The power of linkage detection of each statistic was determined for 2500 replicates of 60 families on chromosome 6, using the thresholds above. The power is 48.5%, 45.9%, and 44.6% for KC-LOD, MLS, and HLOD-S1, respectively, showing the slight advantage of the KC-LOD.

In a second step, we compared the impact of four versus two genetic models in the HLOD analysis. Both statistics have the same the 5% genome-wide threshold. The power of the four-model HLOD-S2 is very slightly, but not significantly, increased (from 44.6 to 45.8%). This increase may be explained by the very strong correlation (*r*^2 ^> 0.97) between the HLODs obtained for the two dominant models (*q *= 0.2 and *q *= 0.01) and for the two recessive models, respectively.

## Conclusion

The linkage statistics studied here are all maximum LOD scores. However, the KC-LOD distribution under the null hypothesis is very different from that of the MLS and HLOD-S1. The same observed value can correspond to very different *p*-values. We would like to stress, following Nyholt [13], that the interpretation of linkage results should not be performed in terms of observed value of the statistics, but that appropriate significance threshold should be empirically calculated on the family structures under study.

It has been claimed that HLOD-S1 had similar or even greater power than so-called nonparametric methods, such as the MLS or the NPL [2]. Here, under the model simulated to mimic HLA susceptibility in rheumatoid arthritis, the power of the KC-LOD is slightly higher than HLOD-S1 and MLS. This result is not general, as it very likely depends on the underlying model and on the sampled family structures. Here, data consisting of affected sib pairs and the information on linkage was only provided by the IBD sharing between affected individuals.

Finally, several authors apply the HLOD statistics, using a wide variety of genetic models, in the hope of better scanning the space of models, and thus increasing the power of detection [6-9]. This is not the case here: performing the analysis under four different genetic models (HLOD-S2) does not increase the power. This is due to the high correlation observed in the value of the statistics under genetic models that differ only by the disease allele frequency. When a LOD score function is maximized over a set of genetic models (the so-called MOD score function [14]), overparameterization may happen for some familial structures [15]. In other words, the same maximum may be reached for an infinite set of key parameters. In particular, Clerget-Darpoux et al. [14] showed that, in nuclear families with two children, the same maximum MOD score was obtained for an infinite set of disease allele frequencies and recombination fractions. Similarly, many sets of disease allele frequency and heterogeneity values can explain the IBD sharing of an affected sib-pair sample.

## Competing interests

The author(s) declare that they have no competing interests.
